# *Clostridioides difficile* Infection (CDI) Disease Burden (Cases, Hospitalizations, and Deaths) in China: A Systematic Literature Review

**DOI:** 10.3390/idr18040067

**Published:** 2026-07-03

**Authors:** Frederick J. Angulo, Genming Zhao, Yuan Wu, Zundong Yin, Jin Yang, Shahnaz Khan, Daniel Khuong Tran, Elisa N. Gonzalez, Anli Sun, Steven Shen, Jamie Findlow

**Affiliations:** 1 Medical Evidence Development, Bacterial Vaccines Real World Evidence and Epidemiology, Pfizer, Collegeville, PA 19426, USA; elisa.n.gonzalez2@pfizer.com; 2 Department of Epidemiology and Biostatistics, School of Public Health, Fudan University, Shanghai 200032, China; gmzhao@shmu.edu.cn; 3 National Institute for Communicable Disease Control and Prevention, Chinese Center for Disease Control and Prevention, Beijing 102206, China; wuyuan@icdc.cn; 4 National Immunization Program, Chinese Center of Disease Control and Prevention, Beijing 100050, China; yinzd@chinacdc.cn; 5 RTI Health Solutions, Durham, NC 27713, USA; yang_jin0104@yahoo.com (J.Y.); skhan@rti.org (S.K.); dtran@rti.org (D.K.T.); 6 Vaccine Medical, Pfizer, Shanghai 200041, China; anli.sun@pfizer.com; 7 Global Vaccines Medical Affairs, Pfizer, Kirkland, QC H9J 2M5, Canada; steven.shen@pfizer.com; 8 Global Vaccines Medical Affairs, Pfizer, Tadworth, Surrey KT20 7NS, UK; jamie.findlow@pfizer.com

**Keywords:** incidence, laboratory-confirmed *Clostridioides difficile* infection, mainland China

## Abstract

Background/Objectives: Although *Clostridioides difficile* infection (CDI) is a key cause of global morbidity and mortality, the burden of CDI in mainland China is not well-defined. The objective of this systematic literature review was to summarize the available epidemiologic evidence on the CDI disease burden (cases, hospitalizations, and deaths) in mainland China. Methods: Six databases (three global [PubMed, Embase, Cochrane] and three Chinese [Chinese National Knowledge Infrastructure, Chinese Science Citation, Wanfang]) were searched on 5 August 2025 using CDI-related and epidemiological search terms. No date or language limits were applied. Real-world epidemiologic studies of adults and/or children with laboratory-confirmed CDI in mainland China reporting population-based CDI incidence, hospital-based CDI incidence, and/or CDI admission rates were included. All studies not meeting these criteria were excluded. Risk-of-bias (RoB) assessment was performed using the Newcastle–Ottawa Scale. Results were summarized descriptively. Results: In total, 11 articles formed the evidence base for this review; each was a single-center, hospital-based study conducted in one of six cities in mainland China and published between 2014 and 2023. RoB assessment indicated that the evidence base was appropriate for this study. No study reported population-based CDI incidence. In total, 10 studies reported hospital-based CDI incidence (0 to 82.0/10,000 patient-days), and four reported CDI admission rates (0 to 23.1/1000 admissions). Eight studies reported mortality rates, which varied across studies. Conclusions: Several single-center, hospital-based studies demonstrate that CDI is present in hospitals in mainland China, but there are no published population-based CDI incidence estimates. These results should be interpreted considering this study’s limitations, including potential publication and selection bias, heterogeneity, and limited generalizability. Overall, the burden of CDI is poorly understood in mainland China. Thus, prospective epidemiological studies, including those with sensitive detection methods, are needed to examine CDI burden across multiple cities in mainland China. These efforts would help illuminate the CDI burden and guide prevention efforts. (PROSPERO ID 1140152; registered 17 March 2026; funding by Pfizer Inc.).

## 1. Introduction

*Clostridioides difficile* infection (CDI) is a leading cause of healthcare- and community-associated infections, bringing about substantial morbidity and mortality globally [1]. With an increasing incidence of infections and associated deaths in recent decades [2,3], CDI represents a critical global public health concern [4]. A high proportion of patients with CDI require hospitalization [5], and, even after appropriate treatment, CDI is frequently recurrent [6]. Patients with CDI typically present with diarrhea and sometimes fever and abdominal pain [7], with symptom severity ranging from mild to severe [1]. The symptoms and potential complications of CDI, including pseudomembranous colitis, toxic megacolon, bowel perforation, sepsis, and organ dysfunction [1], may lead to intensive care unit (ICU) admission and, ultimately, death [8].

CDI diagnosis requires laboratory diagnosis, which relies on appropriate testing of stool samples for toxigenic *C. difficile* and/or the presence of toxin to distinguish CDI cases from other causes of diarrhea [1,9]. Many countries have established public health surveillance for laboratory-confirmed CDI cases [9]. However, most surveillance systems are hospital based and so do not include laboratory-confirmed CDI cases that are not hospitalized [9]. Population-based CDI surveillance programs exist in some countries and provide population-based CDI incidence, hospitalizations, and deaths among individuals who are and are not hospitalized [9,10]. Global estimates of the incidence of healthcare- and community-associated CDI suggest incidence can vary with geographical location, along with other factors, including antibiotic use, older age, and hospitalization [6].

Estimates of CDI disease burden have not been published in China. Available data from a hospital-based study in Hong Kong suggest a lower incidence of hospitalized CDI in China than in the United States. This lower incidence is likely due to underreporting resulting from the underuse of standard-of-care stool specimen collection and testing practices in China [9,11]. However, population-based data from Hong Kong suggest an increase in the incidence of CDI from 2006 to 2014 [11], potentially due to increased stool specimen collection frequency and enhanced laboratory testing practices.

The objective of this systematic literature review (SLR) was to determine the available epidemiologic evidence on the CDI disease burden (cases, hospitalizations, and deaths) in mainland China.

## 2. Materials and Methods

This SLR, including its protocol, was registered on the PROSPERO website (study ID 1140152, registered 17 March 2026; Systematic Literature Review: Epidemiologic Burden of *Clostridium difficile* Infection in China). This SLR was conducted in accordance with Preferred Reporting Items for Systematic Reviews and Meta-Analyses (PRISMA) guidelines [12]. The PRISMA checklist is available in Table S1.

### 2.1. Article Selection Criteria

This SLR identified studies conducted in adults and/or children with laboratory-confirmed CDI in mainland China (i.e., excluding Hong Kong, Macao, Taiwan, and the islands in the South China Sea). Studies included in the data extraction and report were those with real-world epidemiologic data, including retrospective cohort studies (e.g., database analyses, medical record reviews, and registry studies) and prospective observational studies (e.g., cohort studies and case series studies). Outcomes of interest included population-based CDI incidence, hospital-based CDI incidence, and CDI admission rates. All studies not meeting these criteria were excluded prior to data extraction and report. To help identify additional primary studies, SLRs and meta-analyses potentially reporting relevant outcomes were included only for bibliography review but were otherwise excluded.

### 2.2. Databases and Search Strategy

Database searches were conducted on 5 August 2025 using 3 global electronic databases—MEDLINE and MEDLINE In-Process (PubMed platform), Embase (Elsevier Platform), and the Cochrane database—as well as 3 Chinese databases: China National Knowledge Infrastructure (CNKI) database, Chinese Science Citation Database (using Web of Science), and Wanfang Database. Searches consisted of CDI-related terms defining the population, terms restricting studies to mainland China, and terms specifying a broad spectrum of epidemiologic outcomes (i.e., incidence, prevalence, morbidity, and mortality). Search terms contained restrictions to limit returns to studies, including human subjects, and excluded commentaries, letters, editorials, and case reports. Studies on risk factors, prevention, and treatment responses were also excluded. Database-specific syntax was applied in each of the databases (Tables S2–S7). No date limits were applied. The search was not restricted by language.

### 2.3. Screening Process

Records were identified from database searches, duplicate search results were removed using EndNote reference management software (version 25 and above), and the unique references were uploaded to Nested Knowledge (Nested Knowledge Inc., Saint Paul, MN, USA, https://nested-knowledge.com/ (accessed on 1 October 2025, version 1.102.1 and above). Nested Knowledge, an artificial intelligence–powered online literature review platform, was used to manage the screening process. Articles were reviewed by researchers fluent in the published language using the same 2-stage dual-screening process. Disagreements were adjudicated by a senior researcher who was blinded to initial screening decisions. First, the titles and abstracts of unique records were screened, and records not meeting predefined eligibility criteria were excluded. Then, remaining articles advanced to full-text review, where they were further assessed for eligibility. Articles not meeting predefined eligibility criteria were excluded, and the remaining studies formed the evidence base for data extraction and reporting.

### 2.4. Data Extraction and Report

A single researcher manually extracted data from the full-text articles that formed the evidence base, as well as from their associated Supplementary Materials (where available and applicable). Data points extracted from individual studies included reference information (full citation and year of publication) and study characteristics (study objectives, as stated by the authors; study designs; study durations; study periods; descriptions of study populations; data sources; key inclusion and exclusion criteria; hospital/medical center names and descriptions; city/province names; CDI testing methods; and study limitations, as stated by the authors). Baseline characteristics of patients with laboratory-confirmed CDI were also extracted, including specification of adults and/or children in the study, age, sex, key underlying conditions, healthcare setting, duration of hospitalization, key information on antibiotics usage prior to or during CDI, and key information on other medication usage prior to or during CDI. Also, outcomes of CDI epidemiologic burden were extracted: hospital-based incidence, CDI admission rate, population-based incidence, and mortality rate (if reported). All extracted and calculated data points were verified against the primary sources by an independent researcher who was not involved in the data extraction process. Data were tabulated via Excel (version 2408 and above) and summarized descriptively. Meta-analyses and subgroup analyses were neither planned nor feasible given the limited number and heterogeneity of the identified studies.

To compare the results across studies, data from hospital-based studies were expressed as hospital-based CDI incidence (hospitalized CDI cases per 10,000 patient-days) and/or CDI admission rates (hospitalized CDI cases per 1000 hospital admissions). Risk-of-bias assessments were performed by an independent researcher using the Newcastle–Ottawa Scale (a validated tool for appraising non-randomized observational studies [13]), with results confirmed by a second researcher.

## 3. Results

### 3.1. Selection of Articles for Data Extraction and Report

**Figure 1:**
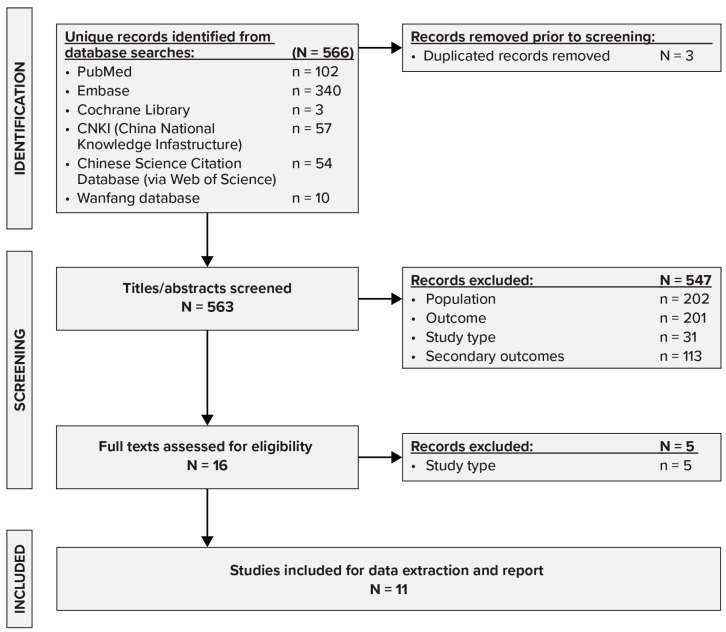
PRISMA diagram. CDI = *Clostridioides difficile* infection; PRISMA = Preferred Reporting Items for Systematic Reviews and Meta-Analyses; SLR = systematic literature review.

A total of 847 references were identified (Table S8); after the removal of duplicates, a total of 563 unique records were included for screening. All 563 articles were written in either English or Chinese. Of the 563 records screened on the basis of their title/abstract, 547 records were excluded. Most records excluded at this step did not match the eligibility criteria in terms of population or outcomes. A total of 16 records advanced to full-text review, five of which were excluded due to study type. At the end of screening, 11 articles were retained for data extraction and reporting and formed the evidence base for this review. Bibliography reviews of SLRs or meta-analyses did not result in identification of additional primary studies. Figure 1 presents the study selection process (i.e., PRISMA diagram).

Based on the risk-of-bias assessment, all 11 studies included for data extraction and report demonstrated a low risk of bias (Figure S1). Despite some limitations, the evidence base was considered sufficiently rigorous to support reliable estimates of CDI incidence in hospital settings in China.

#### 3.1.1. Characteristics of Studies Included in the Data Extraction and Report

All of the 11 studies included for data extraction and report were single-center, hospital-based studies (Table 1). Of these, four were retrospective studies and five were prospective studies, as stated by the authors. One study was described as a case–control study, and one study was described as a cross-sectional study; however, following review of the detailed methodology and results, these two were deemed retrospective in nature. Thus, there were six retrospective studies in total.

**Figure 2:**
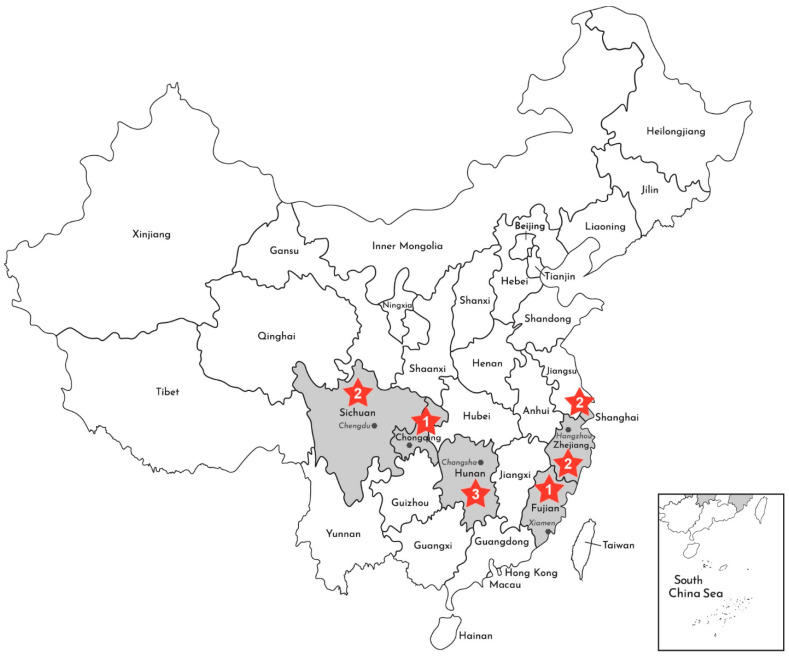
Locations of the studies included for data extraction and report. Note: Numbers in the map indicate number of studies conducted in the marked location.

None of the 11 studies reported population-based CDI incidence, 10 reported hospital-based CDI incidence, and four reported CDI admission rates. Of the 11 studies (Figure 2), three were from Xiangya Hospital in Changsha (Hunan province), two were from the West China Hospital in Chengdu (Sichuan), two were from the First Affiliated Hospital in Hangzhou (Zhejiang), two were from Renji Hospital in Shanghai (direct administered municipality [DAM]), one was from a hospital in Chongqing (DAM), and one was from a hospital in Xiamen (Fujian).

Among the 11 studies included for data extraction and report, publication years ranged from 2014 to 2023, study duration ranged from 4 months to 7 years, and dates of sample collection for patients tested for CDI ranged from 2009 to 2020. Although patient selection varied, all studies involved laboratory-tested stool samples with laboratory confirmation of CDI cases based on 1 or more of the following laboratory tests: stool culture, polymerase chain reaction nucleic acid amplification test (PCR-NAAT), *C. difficile* toxin assay, or mass spectrometry (Supplemental Table S9).

#### 3.1.2. Patient Characteristics

Among the 11 studies included for data extraction and report, the mean age of the study populations ranged from approximately 29 years [15] to 65 years [16]. Most studies were conducted on adults. In two studies that included both adults and children, the populations were mostly adults (age range, 17 to 46 years among patients with CDI in Gu et al. [15] and ≥ 16 years in Zhang et al. [24]). Two of the studies did not specifically state whether the study population included adults or children, although baseline age data suggested they were adults (Qin et al. [20]: mean age of 56.1 years, standard deviation [SD] of 21.7; Dai et al. [14]: mean age of 54.5 years, SD of 17.4). The proportion of males was generally high, ranging from approximately 57% [15] to 83% [16]. Eight of the included studies reported various baseline underlying comorbidities in the study populations, including but not limited to diabetes, hypertension, liver disease, cancer, respiratory failure, neurological diseases, and heart disease [14,15,16,17,18,19,21,22].

All 11 of the studies included for data extraction and report were single-center, hospital-based studies of hospitalized patients. Five studies included only patients hospitalized in ICUs [16,17,19,21,24]. Lengths of stay in the hospital ranged from <2 weeks to >2 months, but the most reported duration was approximately 1 month. Four of the included studies reported information on the duration of hospitalization before CDI onset. Dai et al. [14] reported a median of 7 days from admission to the onset of diarrhea and a median of 14 days from admission to the diagnosis of hospital-acquired CDI. Wang et al. [21] reported a median of 11 days (interquartile range [IQR], 12 days) before CDI. Patients included in Meng et al. [19] had to have diarrhea after 48 h following admission to the hospital. The fourth study, a retrospective study over a 7-year period (2009 to 2016), specifically reported a median of 14 days (IQR, 6–29) of hospitalization prior to CDI onset and a median of 14 days (IQR, 7–31) after CDI onset [22].

The use of antibiotics prior to CDI onset was commonly reported in the studies included for data extraction and report (*n* = 6). Frequently reported antibiotics that patients with CDI received before developing CDI included carbapenems, cephalosporin, β-lactams/β-lactamase inhibitors, and fluoroquinolone [14,15,16,17,21,22]. The use of other medications prior to CDI onset were also reported in six of the selected studies, with proton pump inhibitors, chemotherapies, glucocorticoids, and immunosuppressants being the most commonly reported [14,15,16,17,21,22].

### 3.2. Epidemiologic Burden

**Figure 3:**
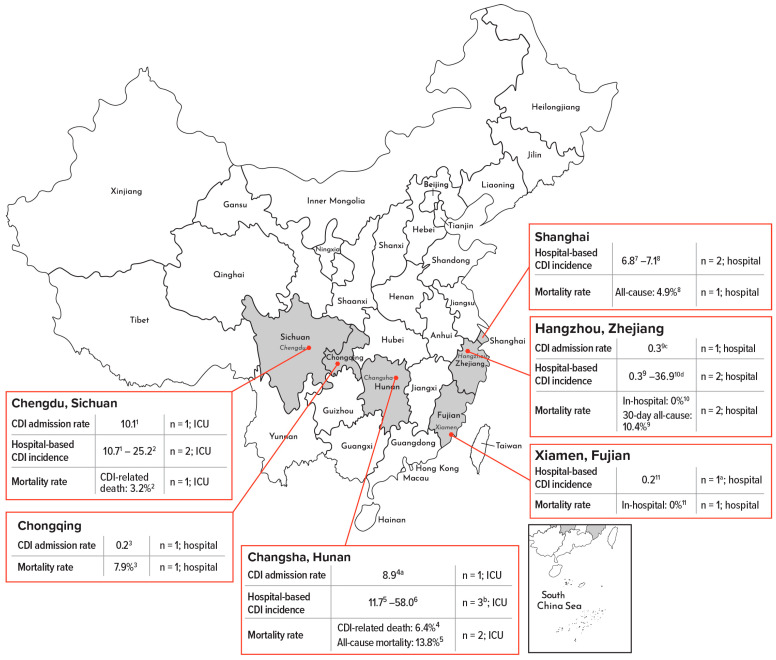
Hospital-based CDI incidence, CDI admission rate, and mortality results by location of the studies included for data extraction and report. CDI = *Clostridioides difficile* infection; HSCT = hematopoietic stem cell transplant; ICU = intensive care unit. Notes: “n” indicates number of studies reporting the relevant outcomes in this location. For CDI admission rate, listed values are number per 1000 admissions. For hospital-based CDI incidence, listed incidence values are per 10,000 patient-days. For mortality rate, listed values are percentages. Sources were as follows: ^1^ Zhang et al. [24]; ^2^ Wang et al. [21]; ^3^ Dai et al. [14]; ^4^ Li et al. [17]; ^5^ Li et al. [16]; ^6^ Meng et al. [19]; ^7^ Qin et al. [20]; ^8^ Yang et al. [23]; ^9^ Xu et al. [22]; ^10^ Gu et al. [15]; ^11^ Ma et al. [18]. ^a^ The listed admission rate is for the overall study population; admission rates in individual specialty ICUs are listed in Table 3. ^b^ The listed incidences are based on data in the overall study populations; incidence data in individual specialty ICUs are listed in Table 2. ^c^ The listed admission rate is the average annual rate over the 85-month study period; this study also reported range of annual admission over the study period (see Table 3). ^d^ The listed incidence is among patients receiving HSCTs, which was the highest value for the purpose of presenting the range in this location; this study also reported incidence among patients receiving chemotherapy (see Table 2).

Figure 3 summarizes the results of hospital-based CDI incidence, CDI admission rate, and CDI mortality rate by locations of the single-center hospitals that conducted each hospital-based study. The three hospitals with results for all three outcomes were in Chengdu (Sichuan province), Changsha (Hunan), and Hangzhou (Zhejiang). Hospital-based CDI incidence and CDI mortality rates were available from hospitals in Xiamen (Fujian) and Shanghai (DAM). CDI admission rates and CDI mortality rates were available from the hospital in Chongqing (DAM).

#### 3.2.1. Hospital-Based CDI Incidence

Hospital-based CDI incidence was reported in 10 of the studies included for data extraction and report, ranging from 0 to 82.0 hospitalized CDI cases per 10,000 patient-days (Figure 3 and Table 2).

**Table 2:** Summary of hospital-based CDI incidence reported by studies included for data extraction and report.

Reference (Year)	Study Design	Healthcare Setting	Patient Population	Location	Duration	Incidence ^a^
Gu et al. [15] (2015)	Retrospective	Hospital	Receiving chemotherapy	First Affiliated Hospital of Zhejiang University; Hangzhou, Zhejiang	4 y	18.9
			Receiving HSCTs			36.9
Xu et al. [22] (2017)	Retrospective	Hospital	Inpatients with confirmed hospital-acquired CDI	First Affiliated Hospital of Zhejiang University; Hangzhou, Zhejiang	7 y (85 mo)	0.3
Qin et al. [20] (2017)	Retrospective	Hospital	Hospitalized patients	Renji Hospital; Shanghai	16 mo	6.8
Yang et al. [23] (2020)	Retrospective	Hospital	Patients with CDI	Renji Hospital; Shanghai	5 y	7.1
Ma et al. [18] (2023)	Cross-sectional (retrospective)	Hospital	Inpatients with loose stool samples	First Affiliated Hospital of Xiamen University; Xiamen, Fujian	4 y	Overall: 0.2 2017: 0.3 2018: 0.3 2019: 0.1 2020: 0.2
Li et al. [16] (2017)	Prospective	ICU	Patients with hospital-acquired pneumonia and hospital-onset diarrhea	Xiangya Hospital; Changsha, Hunan	12 mo	11.7
Li et al. [17] (2018)	Prospective	ICU	ICU patients with ≥ 3 episodes of diarrhea occurring within a 24-h period at least 48 h following admission	Xiangya Hospital; Changsha, Hunan	15 mo	Overall: 14.1 GICU: 17.4 NICU: 21.3 NSICU: 9.9 RICU: 0
Meng et al. [19] (2021)	Prospective	ICU	Hospitalized adults with presumptive antibiotic-associated diarrhea in 4 ICUs	Xiangya Hospital; Changsha, Hunan	8 mo	Overall: 58.0 GICU: 82.0 NICU: 75.7 NSICU: 15.3 RICU: 26.4
Wang et al. [21] (2014)	Prospective	ICU	Patients with ICU-onset diarrhea	West China Hospital; Chengdu, Sichuan	8–9 mo (35 wk)	25.2
Zhang et al. [24] (2016)	Prospective	ICU	Patients with ICU-onset diarrhea	West China Hospital; Chengdu, Sichuan	4 mo	10.7

CDI = *Clostridioides difficile* infection; GICU = general ICU; HSCT = hematopoietic stem cell transplant; ICU = intensive care unit; NICU = neurology ICU; NSICU = neurosurgery ICU; RICU = respiratory ICU. ^a^ Incidence is reported as number of cases per 10,000 patient-days.

Seven studies provided evidence on changes in the hospital-based CDI incidence over time. Ma et al. [18] reported hospital-based CDI incidence from 2017 to 2020 and noted “a downward trend” during this 4-year period. Two prospective studies focused on patients admitted to ICUs and reported detailed, hospital-based CDI incidence results by types of ICU, namely Li et al. [17] and Meng et al. [19]. Although these two studies were conducted by the same research group in the Xiangya Hospital in Changsha (Hunan), the more recent study, Meng et al. [19], reported higher hospital-based CDI incidence both overall and in different specialty ICUs. The authors attributed the higher hospital-based CDI incidence to a more frequent administration of broad-spectrum antibiotics in the most recent years and to more sensitive *C. difficile* detection methods. Another two prospective studies from the West China Hospital in Chengdu (Sichuan) reported differing hospital-based CDI incidence: 25.2 hospitalized CDI cases per 10,000 ICU-days in Wang et al. [21] and 10.7 hospitalized CDI cases per 10,000 ICU-days in Zhang et al. [24]. Zhang et al. [24] attributed the lower incidence to improved infection control practice. From the First Affiliated Hospital of Zhejiang University in Hangzhou (Zhejiang), Xu et al. [22] (2017) reported lower hospital-based CDI incidence compared with Gu et al. [15] (2015); no discussion was provided on this difference.

#### 3.2.2. CDI Admission Rate

CDI admission rates were reported in 4 of the 11 studies included for data extraction and report. The CDI admission rates ranged from 0 to 23.1 hospitalized CDI cases per 1000 admissions (Figure 3 and Table 3). When excluding the zero-case outlier in the respiratory ICU by Li et al. [17], CDI admission rates in the ICU setting were from 4.9 to 23.1 hospitalized CDI cases per 1000 admissions [17,24] and 0.2 to 0.3 hospitalized CDI cases per 1000 admissions in the general hospital setting [14,22] (Table 3).

#### 3.2.3. CDI Mortality Rate

CDI mortality rates were reported in 8 of the 11 studies included for data extraction and report. Overall, CDI mortality rates and associated definitions varied across studies (Figure 3 and Table 4). Two studies reported CDI-related mortality rates, both for patients in the ICU, and rates ranged from 3.2% [21] to 6.4% [17]. The six remaining studies reported mortality rates in patients with *C. difficile*, which may or may not have been due to CDI [14,15,16,18,22,23]. Of these six studies, three reported all-cause mortality (ranging from 4.9% in the hospital setting [23] to 13.8% in the ICU setting [16]), one of which reported 30-day all-cause mortality [22]. The remaining three studies reported in-hospital mortality (0% for both Gu et al. [15] and Ma et al. [18]; 7.9% for Dai et al. [14]).

## 4. Discussion

This SLR was conducted to synthesize evidence on the CDI disease burden (cases, hospitalizations, and deaths) in mainland China. Although 11 single-center, hospital-based studies provided estimates of hospital-based CDI incidence in hospitalized patients with CDI or CDI admission rates, no published estimates of population-based CDI incidence in mainland China were identified in this study. Further, mortality definitions and rates varied across reporting studies. Thus, the burden of CDI remains poorly understood in mainland China. Prospective epidemiological studies using best-practice detection methods are needed to fully understand the CDI burden in mainland China and guide prevention strategies.

In the 11 studies included for data extraction and report, nearly half of the patients were hospitalized in an ICU. The 11 studies reported on patients who were predominantly adult males and patients who had a variety of underlying comorbidities. Length of hospital stay for patients with CDI was typically 1 month; however, because not all studies differentiated between the original reasons for hospitalization (i.e., hospitalization originally due to CDI or hospitalization due to another disease, such as pneumonia [16], liver failure, or malignancy) [21,22], it was not possible to determine the specific length of stay associated with CDI. Information on antibiotic use prior to onset of CDI was reported by 6 of the 11 included studies, but the association between antibiotic use and CDI was not assessed in any of the studies included in this SLR. Many studies have demonstrated that antibiotic use increases the risk of CDI [1]. For example, an SLR and meta-analysis across the Americas, Europe, East Asia, and the Pacific found that the use of third- and fourth-generation cephalosporin and carbapenem antibiotics was associated with an increased risk of CDI in the healthcare setting [25].

The 11 studies forming the evidence base for this SLR reported a wide range of hospital-based CDI incidence, from 0 to 82.0 hospitalized CDI cases per 10,000 patient-days. The most informative study was conducted in collaboration with the United States Centers for Disease Control and Prevention (CDC) in four ICUs at Xiangya Hospital of Central South University in Changsha (Hunan province) and tested the stool samples of all ICU patients with hospital-onset diarrhea using PCR-NAAT [17]. This study found a hospital-based incidence of 14.1 hospitalized CDI cases per 10,000 patient-days [17], which is higher than the 6.9 to 7.6 hospitalized CDI cases per 10,000 patient-days estimated for two medical centers in South Carolina [26].

The evidence for change in hospital-based CDI incidence over time in mainland China was mixed. Hospital-based CDI incidence increased over time in some locations, which was attributed to more sensitive *C. difficile* detection methods (Xiamen) and more frequent administration of broad-spectrum antibiotics (Changsha). By contrast, hospital-based CDI incidence decreased over time in other locations, which was attributed to improved infection control practices (Chengdu). In the First Affiliated Hospital of Zhejiang University in Hangzhou (Zhejiang), the hospital-based CDI incidence was higher in 2015 than in 2017, which may be attributable to the different patient populations included. Gu et al. [15] included patients with underlying compromised immunity (e.g., receiving chemotherapy or hematopoietic stem cell transplantation), whereas Xu et al. [22] included a more general sample of patients.

For the two studies included for data extraction and report that were conducted only among patients in an ICU, CDI admission rates ranged from 4.9 to 23.1 hospitalized CDI cases per 1000 admissions. Overall, CDI admission rates in mainland China were slightly higher than those in the general hospital setting (0.2–0.3 hospitalized CDI cases per 1000 admissions), consistent with a previous meta-analysis across Asia, Europe, and North America that suggested increased prevalence of CDI in the ICU setting [27]. CDI in the ICU context has been associated with increased mortality risk, alongside increased age and the presence of comorbidities, and lower cure rates [28,29]. In the current SLR of studies in mainland China, CDI-related mortality rates were 3.2% to 6.4% in patients hospitalized with CDI (*n* = 2 studies) and 0% to 13.8% in patients hospitalized with *C. difficile* (which may or may not have been due to CDI) (*n* = 6 studies). Taken together, these data underscore that CDI is a considerable public health concern in China.

### Limitations

There are several limitations of this SLR that should be considered when interpreting its results. First, there are limitations inherent to SLRs, including publication bias and study heterogeneity. Although the risk-of-bias assessment indicated that the evidence base was appropriate to address the study objectives, cross-study comparisons should be approached with caution. Given the limited number and heterogeneity of the identified studies, meta-analyses and subgroup analyses were neither planned nor feasible. Study heterogeneity also likely influenced the variation in reported outcomes, such as differences in patient selection, diagnostic algorithms and case definitions, selected denominators, follow-up durations, and outcome definitions (e.g., mortality). It should also be acknowledged that regional variability in both healthcare access and diagnostic capacity may impact these findings. For example, testing density (i.e., frequency of tests relative to the size of the patient population) has been reported in a previous retrospective study to have a large effect on CDI rates [30]. Specifically, low testing density, as well as other factors like the lack of standardized diagnostic methods, can result in CDI burden being underreported [30,31].

This SLR indicated that a limited number of studies have been conducted to assess the burden of CDI in mainland China. Specifically, available evidence comes from six cities, and data are limited to a single hospital in each city, which limit generalizability and may introduce selection bias. For example, the overrepresentation of urban, tertiary-care hospitals likely does not reflect CDI burden in rural or lower-resource settings. Also, the identification of CDI cases relied on standard-of-care stool specimen collection and testing practices, which, as noted above, may differ across hospitals. Still, studies in this SLR consistently demonstrated the presence of CDI in hospitals in mainland China whenever specimens were collected and tested. Importantly, none of the 11 studies reported population-based CDI incidence, an important gap to be addressed in future prospective epidemiological studies. Other important subjects for future research aimed at understanding the CDI disease burden in mainland China include examining healthcare-associated and community-associated CDI and molecular characterization of *C. difficile* isolates from patients with CDI.

## 5. Conclusions

Population-based estimates of the incidence of CDI in mainland China are not available, limiting the evaluation of the CDI disease burden. However, multiple single-center, hospital-based studies demonstrate that CDI is present in hospitals in mainland China. Reported hospital-based CDI incidence ranged from 0 to 82.0 hospitalized CDI cases per 10,000 patient-days, and CDI admission rates ranged from 0 to 23.1 hospitalized CDI cases per 1000 admissions; mortality rates also varied. Still, the burden of CDI is poorly understood in mainland China. Given the limitations of the current available evidence on the CDI disease burden in mainland China, prospective epidemiological studies with sensitive detection methods are needed to further understand the burden of CDI in mainland China and guide prevention efforts.

## Figures and Tables

**Table 1:** Summary of the studies included for data extraction and report.

Reference (Year)	Study Site	Study Design	Dates of Sample Collection for Patients Tested for CDI	Population-Based Incidence	Hospital-Based Incidence	CDI Admission Rate
Dai et al. [14] (2020)	First Affiliated Hospital of Chongqing Medical University; Chongqing	Case–control (retrospective analysis)	June 2014–March 2016	—	—	✓
Gu et al. [15] (2015)	First Affiliated Hospital of Zhejiang University; Hangzhou, Zhejiang	Retrospective	September 2009–August 2013	—	✓	—
Li et al. [16] (2017)	Xiangya Hospital; Changsha, Hunan	Prospective	January 2014–December 2014	—	✓	—
Li et al. [17] (2018)	Xiangya Hospital; Changsha, Hunan	Prospective	June 2013–September 2014	—	✓	✓
Ma et al. [18] (2023)	First Affiliated Hospital of Xiamen University; Xiamen, Fujian	Cross-sectional (retrospective analysis)	January 2017–December 2020	—	✓	✓
Meng et al. [19] (2021)	Xiangya Hospital; Changsha, Hunan	Prospective	April 2017–November 2017	—	✓	—
Qin et al. [20] (2017)	Renji Hospital; Shanghai	Retrospective	May 2014–August 2015	—	✓	—
Wang et al. [21] (2014)	West China Hospital; Chengdu, Sichuan	Prospective	May 2012–January 2013	—	✓	—
Xu et al. [22] (2017)	First Affiliated Hospital, Zhejiang University School of Medicine; Hangzhou, Zhejiang	Retrospective	September 2009–September 2016	—	✓	✓
Yang et al. [23] (2020)	Renji Hospital; Shanghai	Retrospective	September 2014–August 2019	—	✓	—
Zhang et al. [24] (2016)	West China Hospital; Chengdu, Sichuan	Prospective	August 2014–November 2014	—	✓	—

CDI = *Clostridioides difficile* infection. “✓” denotes outcome of interest reported, or that there were data in the article that allowed for calculation of the outcome of interest. “—” denotes outcome of interest not reported in the article, and that no other data could be used to calculate the outcome of interest.

**Table 3:** Summary of CDI admission rate reported in studies included for data extraction and report.

Reference (Year)	Study Design	Setting	Patients	Location	Duration	CDI Admission Rate ^a^
Dai et al. [14] (2020)	Case–control (retrospective)	Hospital	Inpatients with diarrhea	First Affiliated Hospital of Chongqing Medical University; Chongqing	21 mo	0.2
Xu et al. [22] (2017)	Retrospective	Hospital	Inpatients with confirmed hospital-acquired CDI	First Affiliated Hospital of Zhejiang University; Hangzhou, Zhejiang	7 y (85 mo)	0.3 (range, 0.3–0.4 annually over the 85-mo study period)
Li et al. [17] (2018)	Prospective	ICU	ICU patients with ≥3 episodes of diarrhea occurring within a 24-h period at least 48 h following admission	Xiangya Hospital; Changsha, Hunan	15 mo	Overall: 8.9 GICU: 9.7 NICU: 23.1 NSICU: 4.9 RICU: 0
Zhang et al. [24] (2016)	Prospective	ICU	Patients with ICU-onset diarrhea	West China Hospital; Chengdu, Sichuan	4 mo	10.1

CDI = *Clostridioides difficile* infection; GICU = general ICU; ICU = intensive care unit; NICU = neurology ICU; NSICU = neurosurgery ICU; RICU = respiratory ICU. ^a^ CDI admission rate is reported as number of cases per 1000 admissions.

**Table 4:** Summary of mortality rate reported in studies included for data extraction and report.

Reference (Year)	Study Design	Setting	Patients	Location	Duration	Mortality in CDI, *n* (%)
CDI-related mortality rate						
Li et al. [17] (2018)	Prospective	ICU	ICU patients with ≥3 episodes of diarrhea occurring within a 24-h period at least 48 h following admission	Xiangya Hospital; Changsha, Hunan	15 mo	ICU-onset and ICU-associated—3/47 (6.4%) Notes: Three patients died due to CDI (severe diarrhea and toxic megacolon)—6.4%; the other three died from their initial underlying disease (i.e., central nervous system infection, respiratory failure, and chronic obstructive pulmonary disease)—6.4%
Wang et al. [21] (2014)	Prospective	ICU	Patients with ICU-onset diarrhea	West China Hospital; Chengdu, Sichuan	8–9 mo (35 wk)	ICU-onset—1/31 (3.2%) Notes: CDI was considered the attributable cause for one death and contributing cause for another two deaths
Mortality rate of patients with *C. difficile* (may or may not be attributable to CDI)						
Li et al. [16] (2017)	Prospective	ICU	Patients with hospital-acquired pneumonia and hospital-onset diarrhea	Xiangya Hospital; Changsha, Hunan	12 mo	All-cause—4/29 (13.8%) Notes: For deaths in the CDI-positive group, cause of death could not be determined as CDI
Yang et al. [23] (2020)	Retrospective	Hospital	Patients with CDI	Renji Hospital; Shanghai	5 y	All-cause—18 (4.9%)
Xu et al. [22] (2017)	Retrospective	Hospital	Inpatients with confirmed hospital-acquired CDI	First Affiliated Hospital of Zhejiang University; Hangzhou, Zhejiang	7 y (85 mo)	30-day all-cause—32 (10.4%)
Gu et al. [15] (2015)	Retrospective	Hospital	Patients who received chemotherapy or HSCT and had CDI	First Affiliated Hospital of Zhejiang University; Hangzhou, Zhejiang	4 y	In-hospital—0%
Ma et al. [18] (2023)	Cross-sectional (retrospective)	Hospital	Inpatients with loose stool samples	First Affiliated Hospital of Xiamen University; Xiamen, Fujian	4 y	In-hospital—0%
Dai et al. [14] (2020)	Case–control (retrospective)	Hospital	Inpatients with diarrhea	First Affiliated Hospital of Chongqing Medical University; Chongqing	21 mo	In-hospital (unrelated to CDI)—7.9%

CDI = *Clostridioides difficile* infection; HSCT = hematopoietic stem cell transplant; ICU = intensive care unit.

## Data Availability

The original contributions presented in this study are included in the article/Supplementary Materials. Further inquiries can be directed to the corresponding author.

## Supplementary Materials

The following supporting information can be downloaded at: https://www.mdpi.com/article/10.3390/idr18040067/s1. Table S1: PRISMA checklist; Table S2: Embase literature search strategy (5 August 2025); Table S3: PubMed literature search strategy (5 August 2025); Table S4: Cochrane literature search strategy (5 August 2025); Table S5: China National Knowledge Infrastructure literature search strategy (5 August 2025); Table S6: Chinese Science Citation Database (via Web of Science) search strategy (5 August 2025); Table S7: Wanfang literature search strategy (5 August 2025); Table S8: Summary of number of search results; Table S9: Patient selection approaches of the included studies; Figure S1: Summary of risk-of-bias assessment of included studies.

## Abbreviations

The following abbreviations are used in this manuscript:

CDC	Centers for Disease Control and Prevention
CDI	*Clostridioides difficile* infection
CNKI	China National Knowledge Infrastructure
DAM	Direct administered municipality
GICU	General ICU
HSCT	Hematopoietic stem cell transplant
ICU	Intensive care unit
IQR	Interquartile range
NICU	Neurology ICU
NSICU	Neurosurgery ICU
PCR-NAAT	Polymerase chain reaction nucleic acid amplification test
PRISMA	Preferred Reporting Items for Systematic Reviews and Meta-Analyses
RICU	Respiratory ICU
SD	Standard deviation
SLR	Systematic literature review
