# Supplementary material for: Clostridioides difficile Infection (CDI) Disease Burden (Cases, Hospitalizations, and Deaths) in China: A Systematic Literature Review
Source: Infect Dis Rep. 2026 Jul 3;18(4):67. doi: 10.3390/idr18040067 (PMC13397926; doi:10.3390/idr18040067)
Supplement: Supplementary file 1 [file idr-18-00067-s001.zip › idr-4280341-supplementary.pdf]

SUPPLEMENTAL MATERIALS

Table S1. PRISMA Checklist

Section and topic	Item #	Checklist item	Location where item is reported
**Title and abstract**			
Title	1	Identify the report as a systematic review.	Page 1
Abstract	2	See the PRISMA 2020 for Abstracts checklist.	Pages 1-2[a]
**Introduction**			
Rationale	3	Describe the rationale for the review in the context of existing knowledge.	Page 2
Objectives	4	Provide an explicit statement of the objective(s) or question(s) the review addresses.	Page 2
**Methods**			
Eligibility criteria	5	Specify the inclusion and exclusion criteria for the review and how studies were grouped for the syntheses.	Page 3
Information sources	6	Specify all databases, registers, websites, organisations, reference lists and other sources searched or consulted to identify studies. Specify the date when each source was last searched or consulted.	Page 3
Search strategy	7	Present the full search strategies for all databases, registers and websites, including any filters and limits used.	Supplementary Tables S2-S7
Selection process	8	Specify the methods used to decide whether a study met the inclusion criteria of the review, including how many reviewers screened each record and each report retrieved, whether they worked independently, and if applicable, details of automation tools used in the process.	Page 3
Data collection process	9	Specify the methods used to collect data from reports, including how many reviewers collected data from each report, whether they worked independently, any processes for obtaining or confirming data from study investigators, and if applicable, details of automation tools used in the process.	Pages 3-4
Data items	10a	List and define all outcomes for which data were sought. Specify whether all results that were compatible with each outcome domain in each study were sought (e.g., for all measures, timepoints, analyses), and if not, the methods used to decide which results to collect.	Pages 3-4
	10b	List and define all other variables for which data were sought (e.g., participant and intervention characteristics, funding sources). Describe any assumptions made about any missing or unclear information.	Pages 3-4
Study risk of bias assessment	11	Specify the methods used to assess risk of bias in the included studies, including details of the tool(s) used, how many reviewers assessed each study and whether they worked independently, and if applicable, details of automation tools used in the process.	Page 4
Effect measures	12	Specify for each outcome the effect measure(s) (e.g., risk ratio, mean difference) used in the synthesis or presentation of results.	Not applicable
Synthesis methods	13a	Describe the processes used to decide which studies were eligible for each synthesis (e.g., tabulating the study intervention characteristics and comparing against the planned groups for each synthesis (item #5)).	Pages 3-4
	13b	Describe any methods required to prepare the data for presentation or synthesis, such as handling of missing summary statistics, or data conversions.	Page 4
	13c	Describe any methods used to tabulate or visually display results of individual studies and syntheses.	Pages 3-4
	13d	Describe any methods used to synthesize results and provide a rationale for the choice(s). If meta-analysis was performed, describe the model(s), method(s) to identify the presence and extent of statistical heterogeneity, and software package(s) used.	Pages 3-4
	13e	Describe any methods used to explore possible causes of heterogeneity among study results (e.g., subgroup analysis, meta-regression).	Not applicable
	13f	Describe any sensitivity analyses conducted to assess robustness of the synthesized results.	Not applicable

Section and topic	Item #	Checklist item	Location where item is reported
Reporting bias assessment	14	Describe any methods used to assess risk of bias due to missing results in a synthesis (arising from reporting biases).	Not applicable
Certainty assessment	15	Describe any methods used to assess certainty (or confidence) in the body of evidence for an outcome.	Not applicable
**Results**			
Study selection	16a	Describe the results of the search and selection process, from the number of records identified in the search to the number of studies included in the review, ideally using a flow diagram.	Figure 1
	16b	Cite studies that might appear to meet the inclusion criteria, but which were excluded, and explain why they were excluded.	Not applicable
Study characteristics	17	Cite each included study and present its characteristics.	Table 1 and Supplementary Table S9
Risk of bias in studies	18	Present assessments of risk of bias for each included study.	Page 5 and Supplementary Figure S1
Results of individual studies	19	For all outcomes, present, for each study: (a) summary statistics for each group (where appropriate) and (b) an effect estimate and its precision (e.g., confidence/credible interval), ideally using structured tables or plots.	Tables 1-4; there were no effect estimates
Results of syntheses	20a	For each synthesis, briefly summarise the characteristics and risk of bias among contributing studies.	Tables 1-4, page 5, and Supplementary Figure S1
	20b	Present results of all statistical syntheses conducted. If meta-analysis was done, present for each the summary estimate and its precision (e.g., confidence/credible interval) and measures of statistical heterogeneity. If comparing groups, describe the direction of the effect.	Not applicable
	20c	Present results of all investigations of possible causes of heterogeneity among study results.	Not applicable
	20d	Present results of all sensitivity analyses conducted to assess the robustness of the synthesized results.	Not applicable
Reporting biases	21	Present assessments of risk of bias due to missing results (arising from reporting biases) for each synthesis assessed.	Not applicable
Certainty of evidence	22	Present assessments of certainty (or confidence) in the body of evidence for each outcome assessed.	Not applicable
**Discussion**			
Discussion	23a	Provide a general interpretation of the results in the context of other evidence.	Pages 12-13
	23b	Discuss any limitations of the evidence included in the review.	Page 13
	23c	Discuss any limitations of the review processes used.	Page 13
	23d	Discuss implications of the results for practice, policy, and future research.	Pages 12-13
**Other information**			
Registration and protocol	24a	Provide registration information for the review, including register name and registration number, or state that the review was not registered.	Page 2
	24b	Indicate where the review protocol can be accessed, or state that a protocol was not prepared.	Page 2
	24c	Describe and explain any amendments to information provided at registration or in the protocol.	Not applicable
Support	25	Describe sources of financial or non-financial support for the review, and the role of the funders or sponsors in the review.	Page 14
Competing interests	26	Declare any competing interests of review authors.	Page 14
Availability of data, code and other materials	27	Report which of the following are publicly available and where they can be found: template data collection forms; data extracted from included studies; data used for all analyses; analytic code; any other materials used in the review.	Page 14

Note: Based on the 2021 report by Page et al.

[a] The full abstract, included on page 1 of the manuscript, met all criteria in the PRISMA 2020 checklist for abstracts reported by Page et al. (2021).

Table S2. Embase Literature Search Strategy (5 August 2025)

Limits: Humans; no comments, letters, or editorials

Search number	Search terms
Population	
#1	'clostridium difficile infection'/exp OR 'pseudomembranous colitis'/exp
#2	(((clostridium OR clostridioides OR peptoclostridium) NEXT/2 difficile):ti,ab) OR 'c. diff':ti,ab OR 'cdiff':ti,ab OR 'c. difficile':ti,ab OR cdifficile:ti,ab OR (((pseudomembranous OR pseudomembraneous OR pseudomembranacea) NEAR/2 (colitis OR enterocolitis)):ti,ab)
#3	#1 OR #2
China	
#4	'china'/de OR 'guangxi'/exp OR 'inner mongolia'/exp OR 'ningxia'/exp OR 'tibet'/exp OR 'xinjiang'/exp OR 'china':ti,ab,kw,de OR 'chinese':ti,ab,kw,de OR 'china':ff,ad OR 'chinese':ff,ad OR guangxi:ti,ab,kw,de,ff,ad OR 'inner mongolia':ti,ab,kw,de,ff,ad OR 'nei mongol':ti,ab,kw,de,ff,ad OR ningxia:ti,ab,kw,de,ff,ad OR tibet:ti,ab,kw,de,ff,ad OR xizang:ti,ab,kw,de,ff,ad OR xinjiang:ti,ab,kw,de,ff,ad OR sinkiang:ti,ab,kw,de,ff,ad OR 'east turkestan':ti,ab,kw,de,ff,ad
#5	#3 AND #4
Epidemiology	
#6	'epidemiology'/mj OR 'incidence'/exp OR 'prevalence'/exp OR 'morbidity'/exp OR epidemiol*:ti,ab OR inciden*:ti,ab OR prevalen*:ti,ab OR morbid*:ti,ab OR mortal*:ti,ab
#7	#5 AND #6
Exclusions	
#8	'animal'/exp NOT 'human'/exp
#9	comment*:ti OR 'letter':it OR 'editorial':it OR 'case report'/exp OR 'case report*':ti
#10	('risk factor'/exp OR 'prevention'/exp OR 'treatment response'/exp) NOT ('incidence'/exp OR 'prevalence'/exp OR 'morbidity'/exp)
Total	
#11	#7 NOT (#8 OR #9 OR #10)
#12	#11 AND ('article'/it OR 'article in press'/it OR 'erratum'/it OR 'review'/it)

Note: No date or language restrictions were applied.

Table S3. PubMed Literature Search Strategy (5 August 2025)

Limits: Humans; no comments, letters, or editorials

Search number	Search terms
Population	
#1	"Clostridium Infections"[Mesh] OR "Enterocolitis, Pseudomembranous"[Mesh]
#2	"clostridium difficile"[Title/Abstract:~2] OR "clostridioides difficile"[Title/Abstract:~2] OR "peptoclostridium difficile"[Title/Abstract:~2] OR "c diff"[Title/Abstract] OR "cdiff"[Title/Abstract] OR "c difficile"[Title/Abstract] OR cdifficile[Title/Abstract] OR "pseudomembranous colitis"[Title/Abstract:~2] OR "pseudomembraneous colitis"[Title/Abstract:~2] OR "pseudomembranacea colitis"[Title/Abstract:~2] OR "pseudomembranous enterocolitis"[Title/Abstract:~2] OR "pseudomembraneous enterocolitis"[Title/Abstract:~2] OR "pseudomembranacea enterocolitis"[Title/Abstract:~2]
#3	#1 OR #2
China	
#4	"China"[Mesh:NoExp] OR "Tibet"[Mesh] OR china[Text Word] OR chinese[Text Word] OR china[ad] OR chinese[ad] OR guangxi[Text Word] OR guangxi[ad] OR "inner mongolia"[Text Word] OR "inner mongolia"[ad] OR "nei mongol"[Text Word] OR "nei mongol"[ad] OR ningxia[Text Word] OR ningxia[ad] OR tibet[Text Word] OR tibet[ad] OR xizang[Text Word] OR xizang[ad] OR xinjiang[Text Word] OR xinjiang[ad] OR sinkiang[Text Word] OR sinkiang[ad] OR "east turkestan"[Text Word] OR "east turkestan"[ad]
#5	#3 AND #4
Epidemiology	
#6	"Epidemiology"[Majr:NoExp] OR "Incidence"[Mesh] OR "Prevalence"[Mesh] OR "Morbidity"[Mesh] OR epidemiol*[Title/Abstract] OR inciden*[Title/Abstract] OR prevalen*[Title/Abstract] OR morbidit*[Title/Abstract]
#7	#5 AND #6
Exclusions	
#8	"Animals"[Mesh] NOT "Humans"[Mesh]
#9	"Comment"[Publication Type] OR "Letter"[Publication Type] OR "Editorial"[Publication Type] OR "Case Reports"[Publication Type] OR "case report*"[Title]
#10	"Risk Factors"[Mesh] NOT ("Incidence"[Mesh] OR "Prevalence"[Mesh] OR "Morbidity"[Mesh])
Total	
#11	#7 NOT (#8 OR #9 OR #10)

Note: No date or language restrictions were applied.

Table S4. Cochrane Literature Search Strategy (5 August 2025)

Limits: Humans; no comments, letters, or editorials

Search number	Search terms
Population	
#1	[mh “Clostridium Infections”] OR [mh “Enterocolitis, Pseudomembranous”]
#2	(((clostridium OR clostridioides OR peptoclostridium) NEAR/2 difficile):ti,ab) OR “c diff”:ti,ab OR cdiff:ti,ab OR “c difficile”:ti,ab OR cdifficile:ti,ab OR (((pseudomembranous OR pseudomembraneous OR pseudomembranacea) NEAR/2 (colitis OR enterocolitis)):ti,ab)
#3	#1 OR #2
China	
#4	[mh ^”China”] OR [mh “Tibet”] OR china OR chinese OR guangxi OR “inner mongolia” OR “nei mongol” OR ningxia OR tibet OR xizang OR xinjiang OR sinkiang OR “east turkestan”
#5	#3 AND #4
Epidemiology	
#6	[mh ^”Epidemiology”[mj]] OR [mh “Incidence”] OR [mh “Prevalence”] OR [mh “Morbidity”] OR epidemiol*:ti,ab OR inciden*:ti,ab OR prevalen*:ti,ab OR morbidit*:ti,ab
#7	#5 AND #6
Exclusions	
#8	[mh “Animals”] NOT [mh “Humans”]
#9	(Comment OR Letter OR Editorial OR “Case Reports” OR “conference proceeding”):pt OR (case NEXT report*):ti
#10	[mh “Risk Factors”] NOT ([mh “Incidence”] OR [mh “Prevalence”] OR [mh “Morbidity”])
Total	
#11	#7 NOT (#8 OR #9 OR #10)

Notes: No date or language restrictions were applied.

Table S5. China National Knowledge Infrastructure Literature Search Strategy (5 August 2025)

Limits: Humans; no comments, letters, or editorials

Search number	Search terms
#1	(TKA=‘clostridium difficile’ OR TKA=‘clostridioides difficile’ OR TKA=‘peptoclostridium difficile’ OR TKA=‘c diff’ OR TKA=‘cdiff’ OR TKA=‘c difficile’ OR TKA=‘cdifficile’ OR TKA=‘pseudomembranous colitis’ OR TKA=‘pseudomembraneous colitis’ OR TKA=‘pseudomembranacea colitis’ OR TKA=‘pseudomembranous enterocolitis’ OR TKA=‘pseudomembraneous enterocolitis’ OR TKA=‘pseudomembranacea enterocolitis’) AND ((TKA=‘epidemiology’ OR TKA=‘incidence’ OR TKA=‘prevalence’ OR TKA=‘morbidity’) NOT (TKA=‘risk factors’ OR TKA=‘prevention’ OR TKA=‘treatment response’)))<br>academic journals only

Notes: No date or language restrictions were applied.

Table S6. Chinese Science Citation Database (via Web of Science) Search Strategy (5 August 2025)

Limits: Humans; no comments, letters, or editorials

Search number	Search terms
#1	(TS=((((clostridium OR clostridioides OR peptoclostridium) NEAR/2 difficile) OR “c diff” OR cdiff OR “c difficile” OR cdifficile OR ((pseudomembranous OR pseudomembraneous OR pseudomembranacea) NEAR/2 (colitis OR enterocolitis))))) AND TS=((epidemiol* OR inciden* OR prevalen* OR morbidit*) NOT (“risk factor*” OR prevention OR “treatment response”))

Note: No date or language restrictions were applied.

Table S7. Wanfang Literature Search Strategy (5 August 2025)

Limits: Humans; no comments, letters, or editorials

Search number	Search terms
#1	((“clostridium difficile” OR “clostridioides difficile” OR “peptoclostridium difficile” OR “c diff” OR “cdiff” OR “c difficile” OR “cdifficile” OR “pseudomembranous colitis” OR “pseudomembraneous colitis” OR “pseudomembranacea colitis” OR “pseudomembranous enterocolitis” OR “pseudomembraneous enterocolitis” OR “pseudomembranacea enterocolitis”) AND ((“epidemiology” OR “incidence” OR “prevalence” OR “morbidity”) NOT (“risk factor*” OR prevention OR “treatment response”)))

Note: No date or language restrictions were applied.

Table S8. Summary of Number of Search Results

Database type	Database	Retrieved	Unique
Global databases	Embase	343	340
	PubMed	292	102
	Cochrane	10	3
Chinese databases[a]	CNKI	95	57
	Chinese Science Citation Database	72	54
	Wanfang	35	10
	**Total**	**847**	**566**
**Total across all databases after final deduplication**			**563**

CNKI = China National Knowledge Infrastructure.

[a] At the title/abstract level, among the total of 121 unique references from the Chinese databases (57 from CNKI + 54 from the Chinese Science Citation Database + 10 from the Wanfang database), 120 were in Chinese and 1 was in English.

Table S9. Patient Selection Approaches of the Included Studies

Study	Operational CDI definition	Clinical criteria / patient population	Type of stool sample tested	Diagnostic method / algorithm	Distinction between infection and colonization
**Dai (2020)**	Positive toxigenic culture (toxin-producing *C. difficile* strain isolated from a patient with diarrhea).	Hospitalized ≥ 7 days with unexplained diarrhea associated with prior antibiotic administration (AAD).	Unformed stool samples.	Stool anaerobic culture on CCFA; colony confirmation via MS (Vitek MS); *in vitro* toxin A/B detection via ELFA and PCR verification of toxin genes (*tcdA, tcdB, tcdC, cdtA, cdtB*).	**Yes.** Differentiated by requiring the presence of clinical diarrhea symptoms alongside laboratory confirmation of a toxin-producing strain.
**Gu (2015)**	Concurrent positive anaerobic culture and positive toxin-gene assays in a patient with diarrhea.	Hospitalized patients with hematological malignancies undergoing chemotherapy or HSCT who developed diarrhea after admission/while hospitalized.	Semiformed, unformed, or liquid stool samples.	Spore selection via 75% alcohol treatment; anaerobic isolation using CCFA-TA with 7% sheep's blood; MALDI-TOF MS identification; PCR detection of toxin genes (*tcdA, tcdB*) and binary toxin genes (*cdtA, cdtB*).	**Yes.** Confirmed by matching clinical diarrheal symptoms (≥ 2 loose stools within 24 hours after admission) with toxigenic culture results.
**Li (2018)**	Diarrhea presentation coupled with a stool test positive for toxigenic *C. difficile* (*tcdB*-positive) or PMC findings.	Adult patients in 4 non-neonatal ICUs experiencing hospital-onset diarrhea.	Stool specimens.	Anaerobic culture using *C. difficile* CDMN Agar; presumptive identification by colony morphology, horse dung odor, Gram stain, latex agglutination, and PRO DISK; confirmation via 16S rRNA sequence analysis and multiplex PCR for toxin genes (*tcdA, tcdB, cdtA, cdtB*).	**Yes.** Excluded asymptomatic carriers by mandating a strict clinical suspect case definition (≥ 3 loose stools within a 24-hour period occurring ≥ 48 hours post-admission) combined with toxigenic proof.

Study	Operational CDI definition	Clinical criteria / patient population	Type of stool sample tested	Diagnostic method / algorithm	Distinction between infection and colonization
**Li (2017)**	Presence of a confirmed toxigenic *C. difficile* strain in a symptomatic patient's stool sample.	Adult ICU patients diagnosed with hospital-acquired pneumonia or ventilator-associated pneumonia who subsequently developed hospital-onset diarrhea.	Stool samples.	Anaerobic culture on CDMN agar; identification by colony appearance/odor; GDH latex agglutination confirmation; PCR amplification of toxin genes (*tcdA, tcdB, cdtA, cdtB*).	**Yes.** Distinguished active infection from colonization by establishing clinical context (≥ 3 diarrhea episodes within 24 hours occurring ≥ 48 hours after admission) and verifying strain toxigenicity.
**Ma (2023)**	Inpatients presenting with diarrheal symptoms ≥ 48 hours post-admission accompanied by a positive laboratory test result.	Adult inpatients who submitted loose stool samples and had received antibiotic therapy within 8 weeks of diarrhea onset.	Loose stool samples.	Enzyme immunoassay for the simultaneous detection of GDH antigen and free toxins A/B using the *C. diff* Quik Chek Complete assay.	**No / Limited.** Differentiated clinically by tracking loose stools post-admission but noted as a study limitation that formal toxigenic isolate culture/molecular confirmation was not performed, meaning asymptomatic colonization could not be definitively ruled out.
**Meng (2021)**	Positive stool culture for *C. difficile* paired with matching positive toxin-gene detection via PCR in a patient with active clinical symptoms.	Hospitalized adult patients with presumptive AAD in non-neonatal ICUs.	Stool specimens.	Stool sample processing via anaerobic culture on chromogenic CDIF agar (Chrome ID: *C. difficile*); latex agglutination confirmation using GDH testing and PRO DISK; multiplex PCR targeting toxin-producing genes (*tcdA, tcdB, cdtA, cdtB*).	**Yes.** Active infection was verified by ensuring the patient met the clinical definition for AAD (≥ 3 irregular stools per day with Bristol grade 5-7 after 48 hours of admission) alongside toxigenic culture confirmation.

Study	Operational CDI definition	Clinical criteria / patient population	Type of stool sample tested	Diagnostic method / algorithm	Distinction between infection and colonization
**Qin (2017)**	Direct verification of a toxigenic *C. difficile* isolate from a diarrheal stool sample with toxin production verified molecularly.	Inpatients and outpatients with active diarrhea, explicitly excluding those using laxatives or enemas.	Unformed or loose stool samples.	Anaerobic isolation on selective chromogenic CDIF medium; definitive species identification via API 20A and MALDI-TOF MS; PCR verification of toxigenic genes (*tcdA, tcdB, cdtA, cdtB*); total A/B toxin evaluation quantified using automated ELFA (VIDAS).	**Yes.** Colonization was isolated from clinical infection by enforcing a rigorous clinical schema (≥ 3 unformed/loose stools per day for > 3 days without taking laxatives or an enema) paired with confirmation of the isolate's toxigenic profile.
**Wang (2014)**	Diarrhea presentation coupled with a stool sample testing positive for *tcdA* or *tcdB* by direct PCR amplification.	Inpatients in a 50-bed medical ICU who developed nosocomial, ICU-onset diarrhea.	Stool samples.	Total DNA extraction directly from stool; direct screening for pathogenicity locus operon (Paloc) genes *tcdA* and *tcdB* by PCR. Secondary anaerobic culture on CCFA used for isolate verification and MLST.	**Yes.** Active clinical infection was isolated from carriage by strictly selecting patients with ICU-onset diarrhea (≥ 3 loose stools per day for ≥ 1 day occurring > 48 hours after hospital admission).
**Xu (2017)**	Inpatients with diarrhea whose matching stool samples tested positive for both anaerobic culture and toxin-gene assays.	Hospitalized adult patients showing hospital-acquired diarrhea episodes.	Semiformed, unformed, or liquid stool samples.	Anaerobic cultivation on CCFA-TA supplemented with 7% sheep serum; colony verification by MALDI-TOF MS; downstream PCR detection of *tcdA, tcdB, cdtA, and cdtB* genes.	**Yes.** Confirmed as hospital-acquired infection by enforcing clinical symptoms (≥ 3 loose stools within 24 hours established ≥ 48 hours post-admission or within 28 days post-discharge) combined with a confirmed toxigenic profile.
**Yang (2020)**	Presence of consecutive and unformed stools testing positive for toxigenic *C. difficile* or its toxins, or visual confirmation of PMC.	Inpatients and outpatients meeting formal CDI diagnostic protocols across multiple clinical units (e.g., gastroenterology, emergency).	Consecutive unformed stool specimens.	Direct isolation on selective chromogenic CDIF agar; validation by MALDI-TOF MS; PCR identification of housekeeping gene *tpi*, toxin targets *tcdA/tcdB*, and binary toxins *cdtA/cdtB*.	**Yes.** Established true infection over simple microbiological carriage by requiring active diarrhea symptoms paired with confirmation of strain toxigenicity.

Study	Operational CDI definition	Clinical criteria / patient population	Type of stool sample tested	Diagnostic method / algorithm	Distinction between infection and colonization
**Zhang (2016)**	Clinical presentation of watery diarrhea linked to a positive downstream multi-step laboratory validation path for toxigenic *C. difficile*.	Symptomatic inpatients displaying clinical diarrhea as a primary admission diagnosis (community acquired) or secondary manifestation (hospital acquired).	Single fecal samples (watery or unformed stools conforming to Bristol Stool Scale types 6 and 7).	A 3-step algorithm: standard anaerobic cultivation on blood agar and CCFA plates, followed by 16S-PCR confirmation, and final pathogenicity assessment via toxin-gene PCR (*tcdA* and *tcdB*).	**Yes.** Excluded asymptomatic carriers by limiting testing exclusively to individuals presenting with clinical watery diarrhea (≥ 3 unformed episodes within a 24-hour window for at least 1 day).

AAD = antibiotic-associated diarrhea; CCFA = cycloserine-cefoxitin-fructose agar; CDI = *Clostridioides difficile* infection; ELFA = enzyme-linked fluorescent assay; GDH = glutamate dehydrogenase; HSCT = hematopoietic stem cell transplant; ICU = intensive care unit; ID = identifier; MS = mass spectrometry; PCR = polymerase chain reaction; PMC = pseudomembranous colitis; rRNA = ribosomal ribonucleic acid; TA = taurocholate agar.

Figure S1. Summary of Risk of Bias Assessment of Included Studies

First author, year	Design	Selection				Comparability		Outcome / Exposure			Total
		S1	S2	S3	S4	C1	C2	O1 / E1	O2 / E2	O3 / E3	Stars
**Dai 2020**	Case-ctrl	🟢	🟢	🔴	🔴	🟢	🟢	🟢	🟢	🔴	6/9
**Gu 2015**	Case-ctrl	🟢	🟢	🔴	🔴	🟢	🔴	🟢	🟢	🔴	5/9
**Li 2017**	Cohort (pro)	🟢	🟢	🟢	🟢	🟢	🟢	🟢	🟢	🟢	9/9
**Li 2018**	Cohort (pro)	🟢	🟢	🟢	🟢	🟢	🔴	🟢	🟢	🟢	8/9
**Ma 2023**	Cross-sect.	🟢	🟢	🟢	🟢	🟢	🟢	🟢	🔴	🔴	7/9
**Meng 2021**	Cohort (pro)	🟢	🔴	🟢	🟢	🟢	🔴	🟢	🔴	🟢	6/9
**Qin 2017**	Cohort (ret)	🟢	🟢	🟢	🔴	🔴	🔴	🟢	🟢	🟢	6/9
**Wang 2014**	Cohort (pro)	🟢	🟢	🟢	🟢	🟢	🔴	🟢	🔴	🟢	7/9
**Xu 2017**	Cohort (ret)	🟢	🔴	🟢	🟢	🟢	🟢	🟢	🟢	🟢	8/9
**Yang 2020**	Cohort (ret)	🟢	🟢	🟢	🟢	🟢	🟢	🟢	🟢	🔴	8/9
**Zhang 2016**	Cohort (pro)	🟢	🟢	🟢	🟢	🔴	🔴	🟢	🔴	🔴	5/9

C = comparability; CDI = *Clostridioides difficile* infection; Cross-sect = cross-sectional; Ctrl = control; E = exposure (case-control); NOS = Newcastle-Ottawa Scale; O = outcome (cohort); Pro = prospective; Ret = retrospective; S = selection.

Notes: Green circles = low risk (criterion met) and red circles = high risk (criterion not met).

Risk of bias was assessed using the NOS (Wells et al., 2025), which awards up to 9 stars across 3 domains: Selection (4 items), Comparability (2 items), and either Outcome (cohort studies) or Exposure (case-control studies) (3 items). Studies scoring 7 to 9 stars are considered low risk of bias, 4 to 6 are moderate, and 0 to 3 are high. The 2 studies with an explicit case-control design comparing patients with CDI versus non-CDI diarrhea (Dai et al., 2020; Gu et al., 2015) were evaluated using the case-control NOS version; the remaining 9 cohort and cross-sectional studies were evaluated using the cohort NOS.

REFERENCES

Dai W, Yang T, Yan L, Niu S, Zhang C, Sun J, et al. Characteristics of *Clostridium difficile* isolates and the burden of hospital-acquired Clostridium difficile infection in a tertiary teaching hospital in Chongqing, Southwest China. BMC Infect Dis. 2020;20:277. doi:10.1186/s12879-020-05014-6.

Gu SL, Chen YB, Lv T, Zhang XW, Wei ZQ, Shen P, et al. Risk factors, outcomes and epidemiology associated with *Clostridium difficile* infection in patients with haematological malignancies in a tertiary care hospital in China. J Med Microbiol. 2015;64:209-16. doi:10.1099/jmm.0.000028.

Li C, Duan J, Liu S, Meng X, Fu C, Zeng C, et al. Assessing the risk and disease burden of *Clostridium difficile* infection among patients with hospital-acquired pneumonia at a university hospital in Central China. Infection. 2017;45:621-8. doi:10.1007/s15010-017-1024-1.

Li C, Li Y, Huai Y, Liu S, Meng X, Duan J, et al. Incidence and outbreak of healthcare-onset healthcare-associated *Clostridioides difficile* infections among intensive care patients in a large teaching hospital in China. Front Microbiol. 2018;9:566. doi:10.3389/fmicb.2018.00566.

Ma X, Li Z, Zheng Y, Fang L, Lin H, Xu H, et al. A cross-sectional study of *Clostridium difficile* infection in inpatients with antibiotic associated diarrhea. Clin Lab. 2023;69:700-5. doi:10.7754/Clin.Lab.2022.220514.

Meng X, Huang X, Peng Z, Wang Y, Liu S, Zeng C, et al. Antibiotic resistances and molecular characteristics of *Clostridioides difficile* in ICUs in a teaching hospital from Central South China. Front Med (Lausanne). 2021;8. doi:10.3389/fmed.2021.745383.

Page MJ, McKenzie JE, Bossuyt PM, Boutron I, Hoffmann TC, Mulrow CD, et al. The PRISMA 2020 statement: an updated guideline for reporting systematic reviews. Syst Rev. 2021, 10, 89. https://doi.org/10.1186/s13643-021-01626-4.

Qin J, Dai Y, Ma X, Wang Y, Gao Q, Lu H, et al. Nosocomial transmission of *Clostridium difficile* genotype ST81 in a general teaching hospital in China traced by whole genome sequencing. Sci Rep. 2017;7:9627. doi:10.1038/s41598-017-09878-8.

Wang X, Cai L, Yu R, Huang W, Zong Z. ICU-onset *Clostridium difficile* infection in a university hospital in China: a prospective cohort study. PLoS One. 2014;9, e111735. doi:10.1371/journal.pone.0111735.

Wells GA, Shea B, O'Connell D, et al. The Newcastle-Ottawa Scale (NOS) for assessing the quality of nonrandomised studies in meta-analyses. Available online: http://www.ohri.ca/programs/clinical_epidemiology/oxford.asp. Accessed 26 May 2026.

Xu Q, Chen Y, Gu S, Lv T, Zheng B, Shen P, et al. Hospital-acquired *Clostridium difficile* infection in mainland China: a seven-year (2009-2016) retrospective study in a large university hospital. Sci Rep. 2017;7:9645. doi:10.1038/s41598-017-09961-0.

Yang Z, Huang Q, Qin J, Zhang X, Jian Y, Lv H, et al. Molecular epidemiology and risk factors of *Clostridium difficile* ST81 infection in a teaching hospital in Eastern China. Front Cell Infect Microbiol. 2020;10:578098. doi:10.3389/fcimb.2020.578098.

Zhang X, Wang X, Yang J, Liu X, Cai L, Zong Z. Colonization of toxigenic *Clostridium difficile* among ICU patients: a prospective study. BMC Infect Dis. 2016;16:397. doi:10.1186/s12879-016-1729-2.
